# A multi-task graph deep learning model to predict drugs combination of synergy and sensitivity scores

**DOI:** 10.1186/s12859-024-05925-0

**Published:** 2024-10-10

**Authors:** Samar Monem, Aboul Ella Hassanien, Alaa H. Abdel-Hamid

**Affiliations:** 1 Mathematics and Computer Science Department, Faculty of Science, Beni-Suef University, Beni Suef, 62521 Egypt; 2 Faculty of Computer and AI, Cairo University, Cairo, Egypt; 3 Scientific Research School of Egypt (SRSEG), Cairo, Egypt

**Keywords:** Multi-task, Deep learning, Drug combination, Graph network, Task relationships, Attention, Synergy, Sensitivity

## Abstract

**Background:**

Drug combination treatments have proven to be a realistic technique for treating challenging diseases such as cancer by enhancing efficacy and mitigating side effects. To achieve the therapeutic goals of these combinations, it is essential to employ multi-targeted drug combinations, which maximize effectiveness and synergistic effects.

**Results:**

This paper proposes ‘MultiComb’, a multi-task deep learning (MTDL) model designed to simultaneously predict the synergy and sensitivity of drug combinations. The model utilizes a graph convolution network to represent the Simplified Molecular-Input Line-Entry (SMILES) of two drugs, generating their respective features. Also, three fully connected subnetworks extract features of the cancer cell line. These drug and cell line features are then concatenated and processed through an attention mechanism, which outputs two optimized feature representations for the target tasks. The cross-stitch model learns the relationship between these tasks. At last, each learned task feature is fed into fully connected subnetworks to predict the synergy and sensitivity scores.

The proposed model is validated using the O’Neil benchmark dataset, which includes 38 unique drugs combined to form 17,901 drug combination pairs and tested across 37 unique cancer cells. The model’s performance is tested using some metrics like mean square error ($MSE$), mean absolute error ($MAE$), coefficient of determination (${R}^{2}$), Spearman, and Pearson scores. The mean synergy scores of the proposed model are 232.37, 9.59, 0.57, 0.76, and 0.73 for the previous metrics, respectively. Also, the values for mean sensitivity scores are 15.59, 2.74, 0.90, 0.95, and 0.95, respectively.

**Conclusion:**

This paper proposes an MTDL model to predict synergy and sensitivity scores for drug combinations targeting specific cancer cell lines. The MTDL model demonstrates superior performance compared to existing approaches, providing better results.

## Introduction

In challenging diseases, targeting multiple biochemical pathways within a cell with a single drug is often inefficient. The drug combination method, which combines various drugs, offers a potential treatment option with therapeutic effects that surpass the sum of their individual effects. This approach also reduces the risk of side effects by decreasing the required dosage of each drug. Drug combination therapy has been successful for decades, especially in cancer treatment, where drug resistance is prevalent. Consequently, identifying optimal drug combinations is crucial for translational, clinical, and economic research. As a result, sensitivity and synergy are crucial features to consider when evaluating a drug combination.

The drug combination sensitivity is measured by the degree of therapeutic response, commonly expressed as the percentage inhibition of cell viability or growth [1]. Conversely, drug combination synergy refers to the extent of drug interactions that amplify the overall effect of the combined drugs, beyond the individual effects of each drug.

Understanding drug synergy is crucial, as the mechanisms behind synergistic effects are still being explored. Traditional in vivo and in vitro approaches to examining different compounds are time-consuming, costly, and require significant research experience and technical expertise. This is where computational screening of drug synergy becomes vital. By leveraging computational methods, we can efficiently predict and evaluate potential drug synergies, significantly reducing the need for extensive laboratory testing.

To identify synergistic drug combinations, there are two main approaches: in vitro and in vivo experimental approach and in silico computational predictive approach [2]. For in vitro experiments, several established reference models are used to detect synergistic effects, such as Loewe additivity (dose additivity), Bliss independence (Bliss additivity), and the highest single-agent approach. Additionally, the zero-interaction potency model combines the benefits of the Loewe and Bliss models.

Deep learning models play a significant role in biomedical applications, including predicting drug combinations, drug-drug interactions [3], drug toxicity [4], drug-target interactions [5], and so on. These models offer powerful tools for simulating complex biomedical processes, advancing our understanding, and identifying effective drug combinations more efficiently and comprehensively.

This paper proposes an MTDL model named ‘MultiComb’ designed to predict the synergy and sensitivity of drug combinations simultaneously. Initially, drugs are represented by their SMILES structure and processed through a graph convolution network to learn drug features. Concurrently, the cancer cell line is represented by gene expression data using 934 genes. These drug and cell line features are then concatenated into a single feature vector and input into an attention model, to output two different optimized weighted feature vectors for predicting synergy and sensitivity scores. The cross-stitch mechanism is subsequently employed to learn the relationship between the two tasks, facilitating knowledge transfer between them. Finally, the synergy and sensitivity scores are output by learning two fully connected (FC) subnetworks.

The primary contributions proposed by this paper are outlined as follows:

- Learning an MTDL model to predict the two main factors ‘synergy and sensitivity’ to determine the best drug combination for a cancer cell line.
- Graph neural network is presented to extract drug features while FC layers are applied to learn cell line gene expression data.
- An attention mechanism is adapted to customize the shared features for two distinct target tasks.
- The cross-stitch mechanism is applied to learn the relations between these tasks.
- Validating the trained model and outperforming other compares methods.

The following sections of this paper are organized as follows: “Related works” section reviews relevant related works. “The proposed MultiComb model” section presents a detailed explanation of the MultiComb model. “Experimental results” section covers the dataset, regression metrics, and experimental results. Finally, “Results and discussion” section provides the conclusion of the proposed model.

## Related works

Many methods have been proposed to design a simulation model that predicts the efficacy of drug combinations. These methods depend on different aspects including omics data, structure network interaction, and chemical drug properties.

First machine learning algorithms predict drug combinations such as Random Forest, ElasticNet, etc. Then, DeepDSC [6] employed a deep learning model to predict synergistic drug combinations. First, the SMILES feature for drug representation and gene expression features for the cell line are learned, concatenated, and then input into a deep learning model. It achieves higher synergy compared with other machine learning techniques at that moment. The model demonstrated a 7.2% improvement in predicting drug combination synergy compared to five established machine learning techniques.

After that, AuDNNsynergy [7] uses omics data with a deep learning model. This proposed method assumed that the cell line feature consists of three components: gene expression, copy number, and genetic mutation data. So, three autoencoders are used to represent features for each component. Then, the drug features were merged with the cell line features and input to a deep learning model. Using omics data with deep learning outperforms DeepDSC, which only depends on a deep learning model.

In 2021, MatchMaker [8] proposed an enhanced deep learning model which consists of two parallel networks. First MatchMaker extracts cell line features from gene expression data and SMILES fingerprint for drugs and concatenates the feature cell with each drug feature individually. Then, the two inputs are fed to two parallel subnetworks. After that, the outputs of the two subnetworks are concatenated and fed to another subnetwork to predict the synergy score. This model achieved a 4.0% improvement in $MSE$ compared to AuDNNsynergy. Also, this paper shows that the MatchMaker model can predict sensitivity scores for drug combinations.

All the previous methods used a reference model to calculate the synergy. The reference model is a model that uses the ‘Loewe additivity score’ that assumes the drugs are not combined with itself. SynPred [9] model used more than one reference model, such as Loewe additivity mode, Bliss independence model, highest single agent model, and Zero interaction potency model. It used the full omics data of cell lines and the chemical descriptor for drugs. It used an autoencoder for each cell line feature (Gene expression, Copy Number Variation, Methylation, Global Chromatin Profiling, Metabolomics, microRNA, and Proteomics) to reduce the dimension of omics data. It used the ‘Mordred’ function for drug features to get 1613 features for 43 different chemical classes. Then, the data is fed to a deep learning model. According to [10], SynPred achieved a high precision score over DeepDSC and AuDNNsynergy.

The work in TranSynergy [11] proposed a method that depends on the cellular effect of drug actions which can be modeled by gene dependencies and gene–gene interactions. proposed an innovative approach known as Shapley Additive Gene Set Enrichment Analysis to identify the genes that enhance the synergy of drug combinations. The drug features are extracted from 2041 selected genes by drug-target interaction and cell line features are extracted from the 2041 gene dependency or gene expression. Finally, these features are fed to a deep learning model designed based on a transformer.

In DeepDDS [12], the work proposed a new method for representing drug features. Drugs were represented as graphs, where atoms served as nodes and chemical bonds as edges. A graph neural network is then employed t to extract the drug’s molecular features. Additionally, cell line features are derived from gene expression data, with a multi-layer perceptron used to process these features. The drug and cell line features are concatenated into a single vector, which is then input into fully connected layers to predict whether a drug combination is synergistic or antagonistic based on the synergy value. The published results show that DeepDDS outperforms TranSynergy and DeepDSC. All the previously discussed deep learning works are summarized in Table 1.

**Table 1:** The summarization of the related works

	Main features of drugs	Main features of cell line	Deep learning model	Output
DeepDSC [6]	Used drug SMILES to extract drug features	Gene expression data is used to create a feature vector for the cell line	Processes the concatenated vector of drug and cell line features through three fully connected layers	Loewe synergy score
AuDNNsynergy [7]	Used drug SMILES to extract drug features	Gene expression, copy number, and genetic mutation data are used to create a comprehensive feature vector	Processes the drug and cell line features by passing them through fully connected layers	Loewe synergy score
SynPred [9]	Used drug SMILES to extract drug features	Utilized gene expression, copy number variation, methylation, global chromatin profiling, metabolomics, microRNA, and proteomics features	Utilized a fully connected subnetwork to integrate cell line features with the features of the two drugs	Loewe synergy, Bliss synergy, highest single agent model, and Zero interaction potency model
MatchMaker [8]	Used drug SMILES to extract drug features	Gene expression data is used to create a feature vector for the cell line	Concatenate each drug feature with the cell line feature and pass the combined data through two parallel fully connected subnetworks. The outputs of these subnetworks are then concatenated and fed into a final fully connected subnetwork	Sensitivity and synergy scores
TranSynergy [11]	Drug features extracted from 2041 selected genes by drug-target interaction	Cell line features extracted from 2041 gene dependency or gene expression	Used a transformer deep learning model to map concatenated drug and cell line features	Loewe synergy score
DeepDDS [12]	Convert the drug SMILES to a graph network	Used gene expression of cell line as a feature vector	Used a graph attention network for drugs and fully connected subnetwork for cell lines then concatenate them and fed to other fully connected layers	Binary classification of drugs (synergistic or antagonistic)

All previous related works treat synergy and sensitivity scores as independent tasks. However, synergy and sensitivity scores are inherently related. By transferring knowledge and learning the relationships between these tasks, the accuracy of the prediction model can be improved. Therefore, this paper introduces an MTDL model to enhance the prediction of synergy and sensitivity values for drug combinations. This is achieved by optimizing shared features and transferring knowledge between the drug combination metrics (synergy and sensitivity).

## The proposed Multicomb model

**Fig. 1:**
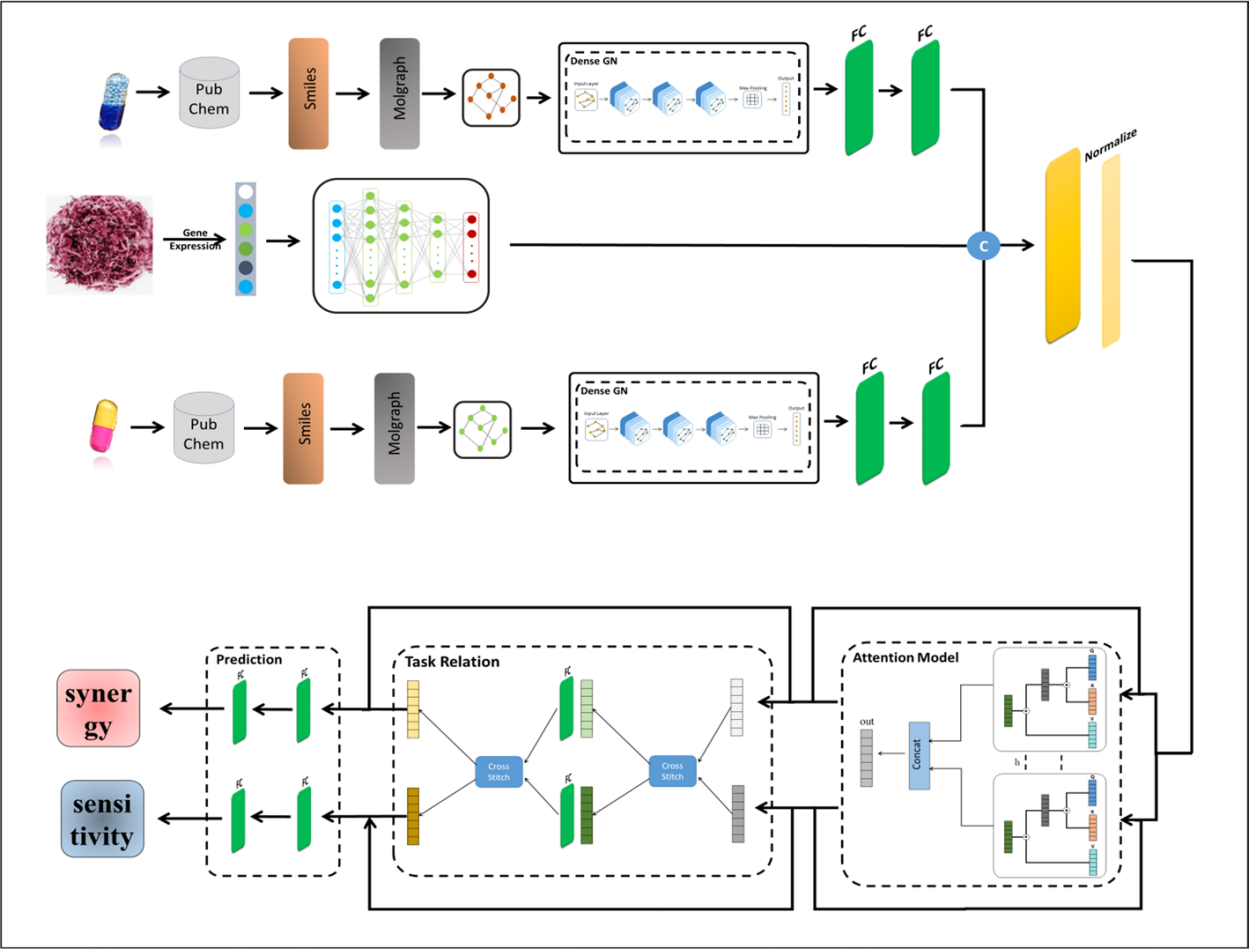
The structure of the MultiComb model

This section discusses the proposed MultiComb model to predict drug combinations’ synergy and sensitivity scores at a cell line. As mentioned before, the MultiComb model is an end-to-end MTDL model. Figure 1 illustrates the structure of the MultiComb model. First, features for both drugs and cell lines are extracted. Then, a network is designed to simultaneously predict the synergy and sensitivity score. “Data representation” subsection discusses the data representation, while “Graph convolution network” subsection discusses the graph convolution network. After that, “Combined cell and drug features” subsection discusses combining drugs and cell line features. Finally, “Attention model” and “Cross-stitch subnetwork” subsections discuss the attention and cross-stitch networks.

### Data representation

First, we extract the SMILES of drugs from the PubChem website to process the drug features. Then, the drug molecular graph is extracted from drug SMILES by the freely open-source chemical informatics package RDKit. This drug molecular graph comprises nodes that represent atoms and edges that denote chemical bonds. This graph can be formally as $G = (N;E)$, where $N$ denotes the set of $n$ nodes each represented as a $K-dimensional$ feature vector with $K$ feature vector length. $E$ represents the set of edges that can be defined by an adjacency matrix $A$. In the molecular graph, the $i-th$ atom is represented by ${a}_{i} \epsilon \in N$, and the chemical bonding between the $i-th$ atom and $j-th$ atom is represented by ${e}_{ij}\in E$. The drug features extracted from a molecular graph are non-Euclidean features, although not translation-invariant. So, a graph neural network is applied rather than a standard convolution network.

The DeepChem [13] is an open-source library designed for applying deep learning to problems in chemistry, materials science, and biology. Within DeepChem, the MolGraphConvFeaturizer [14] tool converts molecular structures into numerical representations suitable for graph-based deep learning models. The MolGraphConvFeaturizer extracts a comprehensive set of features that effectively capture the structural and chemical properties of molecules, making it a powerful tool for representing drugs. It computes a binary vector for each atom in a molecule, capturing essential properties such as atom type, hydrogen bonding type (donor or acceptor), hybridization (‘sp’, ‘sp2’, ‘sp3’), aromaticity, degree of the atom (ranging from 0 to 5), formal charge, and the number of connected hydrogens (ranging from 0 to 4). These features enable effective training of machine learning models on molecular data and summarized in Table 2.

**Table 2:** The summarization of MolGraphConvFeaturizer features

Feature	Description	Length
Atom type	A one-hot encoded vector representing the atom type, such as “C”, “N”, “O”, “F”, “P”, “S”, “Cl”, “Br”, “I”, or other atoms	10
Formal charge	An integer value represents the electronic charge of the atom	1
Hybridization	A one-hot encoded vector specifying the hybridization state of the atom: “sp”, “sp2”, or “sp3”	3
Hydrogen bonding	A one-hot encoded vector represents whether the atom is a hydrogen bond donor or acceptor	2
Aromatic	A one-hot encoded vector that shows whether the atom is part of an aromatic ring	2
Degree	A one-hot encoded vector representing the atom’s degree, ranging from 0 to 5	6
Number of Hydrogens	A one-hot encoded vector indicating the number of hydrogens (0–4) bonded to the atom	5
Chirality	A one-hot encoded vector denoting the chirality of the atom: “R” or “S”	2

To represent the cell lines, gene expression data is used. This data is obtained from the Cancer Cell Line Encyclopedia (CCLE) [15], a project dedicated to studying cancer cell lines’ genomes, microRNA expression, and anticancer drug dose responses. The CCLE gene expression data contains 57,820 gene features per cell line.

As observed, there is an imbalance in the dimensional data between drugs and cancer cell line features. To address this, the dimensionality of the cancer cell line features is reduced using the LINCS [16] project. The LINCS project identified a set of 1000 carefully selected genes, known as the ‘Landmark gene set,’ that can capture more than 80% of the characteristics of any cancer cell line based on connectivity map data. Subsequently, the genes that intersect between the CCLE gene expression data and the Landmark gene set are selected. Ultimately, 934 genes are selected to represent the final cell line vector, which is then normalized by the tanh-norm method.

### Graph convolution network

A graph convolution neural network learns the drug features. The message is transferred between each atom and its adjacent atoms in the learning process. In this paper, the graph convolution network (GCN) [17] is trained as a graph network in the learning framework to extract drug features.

The GCN model utilizes an effective layer-wise propagation mechanism. This layer propagation mechanism can be represented in Eq. (1). The initial input to the GCN consists of the atom properties matrix $X\in {R}_{n\times 30}$ and the adjacency matrix $A\in {R}_{n\times n}$. In the adjacency matrix, ${e}_{ij}\in A$ is equal to 1 if there is a chemical bond between $i-th$ and $j-th$ atoms; otherwise is equal to 0.

1 $$\grave{X}_{{\text{i}}} = \alpha WX_{i} + b,$$

where $W$ and $b$ are learned weight and biased, and $\alpha$ is calculated as in Eq. (2).

2 $$\alpha ={\widehat{D}}^{-1\backslash 2} \widehat{A} {\widehat{D}}^{-1\backslash 2},$$

where $\widehat{A}=A+I$ is the modified graph adjacency matrix with self-loops added, $I$ represents the identity matrix and $\widehat{D}$ denotes the degree matrix of the modified adjacency matrix. After each layer, a LeakyReLU activation function and dropout connections are applied to the final output $\grave{X}$.

Also, a regularization method is applied to the weights and outputs of layers to reduce overfitting between training and testing scores. a global max pooling layer is applied to the output of the final graph layer to transform the ${\text{X}}_{\text{i}}$ matrix to a featured vector for the next steps.

### Combined cell and drug features

First, the subnetwork is learned to extract the features of the cell line. The subnetwork consists of three FC layers, each followed by an activation function and a dropout layer. ReLU is used as the activation function following each FC layer, with a dropout rate of 0.2 applied after both the first and second FC layers. However, no dropout follows the final FC layer. In this paper, the FC layer is regularized by applying techniques to both the weights and outputs of the FC layer to prevent overfitting of the learning model. The first FC layer of the subnetwork receives the gene expression data of the cancer cell line. The FC layers then learn the cell line features, producing the final feature vector of the cancer cell line.

On the other hand, the drug feature vector output from the GCN is passed through two fully connected layers with ReLU activation, without dropout, to produce the final drug feature vector. This vector is then concatenated with the cell line feature vectors into a single combined vector. A normalization layer is applied after concatenation to maintain the integrity of the combined cell line and drug features. These normalized data are then used as input for the attention model.

### Attention model

An attention model [18] is applied to outperform different task feature representations. Also, attention gates are used to guide the learning process of various tasks to focus on the extracted features completely. In this paper, multi-head attention is used. Multi-head attention based on weighting extracted features for different tasks separately. So, it focuses on the important features and minimizes the noise.

Multi-attention consists of three main inputs: query ($q$), key ($k$), and value ($v$) vectors. Here, for each task, the attention mechanism maps the normalized concatenation layer ${\prime}{h}_{0}{\prime}$ to $q$, $k$, $v$ using distinct linear projection layers, as shown in Eqs. (3), (4), and (5).

3 $${h}_{q}=f\left(wa*h+b\right),$$

4 $${h}_{k}=f\left(wa*h+b\right),$$

5 $${h}_{v}=f\left(wa*h+b\right),$$

where $f$ is the activation function of attention while $wa$ and $b$ are the learning attention weight and bias vectors, respectively.

After that, the dot-product is applied to them as represented in Eq. (6).

6 $$s=softmax\left({h}_{q}{*h}_{k}\right),$$

Finally, the cell line vector is summarized in Eq. (7).

7 $${h}_{f}^{1}=\sum s*{(h}_{v}* {W}_{s}),$$

where ${W}_{s}$ is the scale weight of the cell line feature.

This process is repeated across $h$ parallel attention heads, and the resulting vectors from each head are concatenated to produce the final vector, as described in Eq. (8). Here, $h$ is set to 4.

8 $${h}_{f}=concat\left({h}_{f}^{1},{h}_{f}^{2},\dots \dots ,{h}_{f}^{h}\right).$$

Finally, the output from the multi-head attention mechanism is concatenated with its input according to Eq. (9).

9 $${h}_{ff}=concat\left({h}_{f},{h}_{0}\right).$$

### Cross-stitch subnetwork

One major factor in the MTDL model is determining the relationships between tasks. The correct task relationship setting can enhance overall task performance. On the other hand, inaccurate relationship setup may lead to negative knowledge transfer and decrease prediction accuracy. So, in this paper, the cross-stitch subnetwork [19] is implemented to discover the relationships between synergy and sensitivity tasks.

The cross-stitch learns the task relationships during the training of the MultiComb model. It uses the cross-stitch unit to determine the extent of information sharing needed. The cross-stitch function is defined by Eq. (10).

10 $$\left[{\overline{a} }_{1} {\overline{a} }_{2}\right]=\left[\begin{array}{cc}{k}_{11}& {k}_{12}\\ {k}_{21}& {k}_{22}\end{array}\right] \left[{a}_{1} {a}_{2}\right],$$

where ${a}_{1}$ and ${a}_{2}$ represent the first and second input task features, respectively, while ${\overline{a} }_{1}$ and ${\overline{a} }_{2}$ represent the task relationship features for the first and second tasks, respectively. The value ${k}_{ij}$ represents the of the weighted relationship between tasks $i$ and $j$.

In this paper, the final output from the multi-head attention mechanism for each task is fed into the cross-stitch network. according to Eq. (11).

11 $$\left({\overline{h} }_{ff1},{\overline{h} }_{ff2}\right)=cros{s}_{stitch\left({h}_{ff1},{h}_{ff2}\right)},$$

where, ${h}_{ff1} and {h}_{ff2}$ represent the multi-head attention features for synergy and sensitivity tasks separately.

Then, the output vectors ${\overline{h} }_{ff1}$ and ${\overline{h} }_{ff2}$ from the cross-stitch network are then passed through an FC layer separately, resulting in new representations ${h}_{s1}$ and ${h}_{s2}$ for each task, respectively.

Following this, an additional cross-stitch layer is added as described by Eq. (12).

12 $$({\overline{h} }_{s1},{\overline{h} }_{s2})=cross\_stitch({h}_{s1},{h}_{s2}),$$

Finally, the inputs and outputs of the cross-stitch layer are concatenated according to Eqs. (13) and (14).

13 $${h}_{sf1}=concat\left({\overline{h} }_{s1},{h}_{ff1}\right),$$

14 $${h}_{sf2}=concat\left({\overline{h} }_{s2},{h}_{ff2}\right).$$

After capturing the two tasks’ relationship features, the prediction scores are learned by a prediction subnetwork. The prediction subnetwork consists of two FC layers, and each task feature is fed to a separate subnetwork and outputs the synergy and sensitivity scores.

## Experimental results

The MultiComb model is evaluated using one of the challenging O’Neil datasets [20]. This section begins with an overview of the dataset, followed by a discussion of the evaluation metrics in the subsequent subsection. The final subsection presents and analyzes the experimental results of the MultiComb model applied to the dataset.

### Dataset characteristics and analysis

The drug combination data is obtained from the extensive O’Neil cancer screening dataset. This dataset includes the names of the two drugs being combined and the targeted cancer cell line. It contains data on 38 unique drugs and their interactions, resulting in a large number of drug combination pairs tested across 39 cancer cell lines. Specifically, the dataset includes 23,052 drug combination pairs. So, the dataset consists of 38 unique drugs that are combined to get 23,052 drug combinations. These drug combinations are tested on 39 cancer cell lines. The dataset spans seven cancer tissue types: skin, ovary, lung, large intestine, breast, prostate, and pleura. To evaluate the pharmacological impact of these combinations, the Loewe Additivity score [21] is calculated, determining whether the combination has a synergistic or antagonistic effect. This score is derived from a 4 by 4 dose–response matrix and assumes no interaction between the drug and itself.

The synergy scores in the O’Neil cancer dataset range from − 326.464 to 179.1233. In this dataset, a single drug combination may be evaluated multiple times. Consequently, for every distinct drug pair-cell line combination, the target synergy scores are determined by averaging the replicate scores.

On the other hand, the sensitivity scores for drug combinations are obtained from [1]. These scores are calculated using the area under dose–response curves, with values ranging from 0 to 46.6657. For training the MultiComb model, the intersecting values from the synergy and sensitivity scores for each unique drug combination are selected. This process results in a benchmark dataset comprising 17,901 unique drug combinations, involving 38 distinct drugs and 37 cancer cell lines.

In this paper, the O’Neil dataset is randomly split into five cross-fold validations, ensuring that each drug-drug combination pair appears in only one fold, as suggested in [6]. This approach ensures that drug-drug combination pairs in the testing set do not appear in the training set. Additionally, the dataset is augmented by reversing the order of the input drug pairs. Each fold is used once as the testing dataset, while the remaining four folds serve as the training dataset for the model. Finally, the mean synergy and sensitivity values are reported across all five training iterations.

### Evaluation metrics

Five regression metrics are employed to evaluate the performance of the MultiComb model. The first metric is the mean squared error (*MSE*), which calculates the squared difference error between the actual and predicted scores, as defined in Eq. (15).

15 $$MSE=\frac{\sum_{i=1}^{n}{ ({y}_{a}^{i}-{y}_{p}^{i})}^{2}}{n},$$

where ${y}_{a}^{i}$ and ${y}_{p}^{i}$ specified the actual and predicted scores, respectively and $n$ represents the total number of samples. Also, the root means square error ($RMSE$) is used, equal to the root of $MSE$. The *MSE’s* 95% confidence interval is computed and presented. The second metric is a mean absolute error ($MAE$), also used to determine the difference between the actual and predicted scores. However, $MAE$ differs from $MSE$ as $MSE$ assigns high weights to predicted error values. Equation (16) shows the $MAE$.

16 $$MAE= \frac{\sum_{i=1}^{n}\left|{y}_{a}^{i}-{y}_{p}^{i}\right|}{n}.$$

The third metric is the coefficient of determination (${R}^{2}$) which measures the goodness of fit for the prediction model. ${R}^{2}$ is shown in Eq. (17).

17 $${R}^{2}=1-\frac{\left|MSE\right|}{var\left({y}_{a}\right)},$$

where

18 $$var\left({y}_{a}\right)=\frac{\sum_{i=1}^{n}{\left({y}_{a}^{i}-{\overline{y} }_{a}^{i}\right)}^{2}}{n}.$$

The ${R}^{2}$ value ranges from 0 to 1, where 0 indicates a poor prediction model and 1 indicates a perfect prediction model.

The fourth metric is the Pearson correlation coefficient (${CC}_{P}$) which assesses the variability and consistency between actual and predicted values. The calculation for ${CC}_{p}$ is provided in the following Eq. (19).

19 $${CC}_{P}=\frac{\sum_{i=1}^{n}({y}_{a}^{i}-{\overline{y} }_{a})(({y}_{p}^{i}-{\overline{y} }_{p})}{\sqrt{\sum_{i=1}^{n}{({y}_{a}^{i}-{\overline{y} }_{a})}^{2}} \sqrt{\sum_{i=1}^{n}{({y}_{p}^{i}-{\overline{y} }_{p})}^{2}}},$$

where ${\overline{y} }_{a}$ and ${\overline{y} }_{p}$ indicate the mean value of actual and predicted values, respectively. ${CC}_{p}$ ranges from − 1 to 1, with a higher value indicating a stronger correlation between the actual and predicted values.

The fifth metric is the Spearman correlation coefficient (${CC}_{s}$) which indicates both the direction and degree of the relationship between actual and predicted values. Equation (20) shows the calculation for ${CC}_{s}$.

20 $${CC}_{S}={CC}_{p}(R\left({y}_{a}\right),\left(R\left({y}_{p}\right)\right),$$

where $R\left({y}_{a}\right)$ and $R\left({y}_{p}\right)$ represent the rank values of the actual and predicted scores, respectively.

Hyperparameter training settings completely define the architecture of the MultiComb model. These settings are fixed for all the related works being compared to ensure a fair comparison. During the training of the MultiComb, the learning rate ‘lr’ of the training process is set to 0.1e−4, and the Adam optimizer is applied to optimize the model’s weights. The maximum number of epochs for training is set at 500, with a batch size of 64. A dropout rate of 0.2 is applied, and $MSE$ is used as a loss function.

Another important hyperparameter is identified in the number of neurons in the GCN network layers, which are configured with 78, 156, and 312 units, respectively. Also, the units of each three FC layers that extracted the cell line features are set to 512, 256, and 128, respectively.

## Results and discussion

Table 3 shows the experimental results obtained from the testing dataset to predict synergy using the evaluation regression metrics between MultiComb and other compared methods. MultiComb achieves the lowest $MSE$, $RMSE$, and $MAE$. Also, MultiComb achieves the highest ${CC}_{p}$, ${R}^{2}$, and ${CC}_{s}$. The $MSE$ of the MultiComb model is 232.37, which is lower than that of DeepDSC by 25.37, MatchMaker by 21.75, and DeepDDS by 15.83. Also, MultiComb enhances $MAE$ with 5.80% for DeepDSC, 5.24% for MatchMaker, and 3.90% for DeepDDS. This indicates that the MultiComb model can predict accurate synergy scores well.

**Table 3:** Comparison of MultiComb with other algorithms for predicting synergy scores

Performance metrics	MSE		RMSE		MAE		${R}^{2}$		${CC}_{p}$		${CC}_{s}$	
	Mean	Std	Mean	Std	Mean	Std	Mean	Std	Mean	Std	Mean	Std
DeepDSC	257.74	41.98	16.00	1.28	10.18	0.56	0.52	0.04	0.72	0.03	0.71	0.02
MatchMaker	254.12	40.60	15.93	1.24	10.12	0.05	0.53	0.03	0.73	0.02	0.71	0.02
DeepDDS	248.20	45.56	15.69	1.41	9.98	0.58	0.54	0.05	0.74	0.03	0.71	0.02
MultiComb	**232.37**	**41.24**	**15.19**	**1.32**	**9.59**	**0.52**	**0.57**	**0.04**	**0.76**	**0.02**	**0.73**	**0.02**

The best-performing results are shown in bold

Also, the ${R}^{2}$ score of MultiComb for synergy is higher than DeepDSC by + 0.05, MatchMaker by + 0.04%, and DeepDDS by + 0.03. So, the MultiComb model performance is good compared to other models. For the ${CC}_{P}$ metric, MultiComb outperforms DeepDSC by + 0.04, MatchMaker by + 0.03%, and DeepDSC by + 0.02. This indicates that the consistency of MultiComb can be acceptable. For the last metric ${CC}_{s}$_,_ we can see the MultiComb enhanced ${CC}_{s}$ over DeepDSC, MatchMaker, and DeepDDS by 0.02 indicating the strongest relation between predicted synergy and actual synergy.

**Fig. 2:**
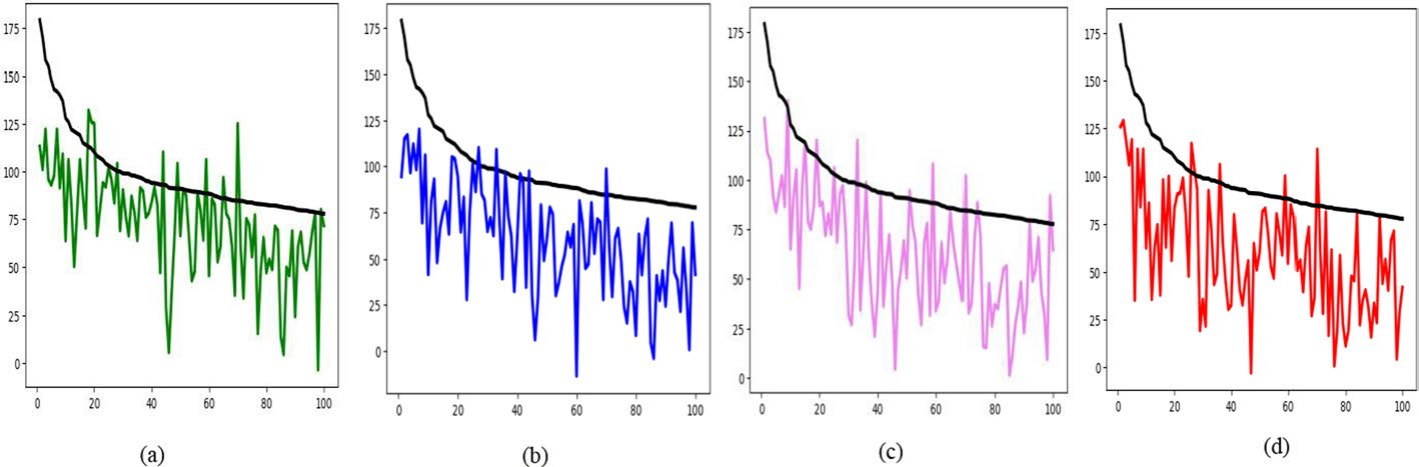
The top 100 actual synergy score versus predicted synergy scores for **a** MultiComb, **b** DeepDDS, **c** MatchMaker, and **d** DeepDSC models

Also, in Fig. 2, the top 100 synergy scores are visualized against the predicted scores for each model: MultiComb, DeepDDS, MatchMaker, and DeepDSC. The black line represents the actual synergy scores, while the colored line represents the predicted synergy scores. As shown, the MultiComb model’s line intersects the black line the most and shows less variation between the black and colored lines compared to the other models.

**Fig. 3:**
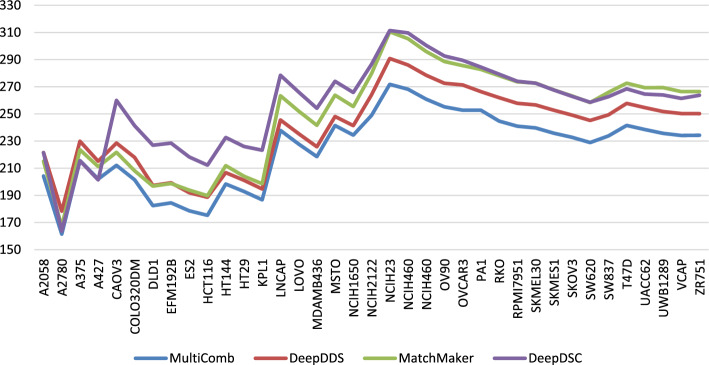
The performance of the MSE for different methods in each cancer cell line

**Fig. 4:**
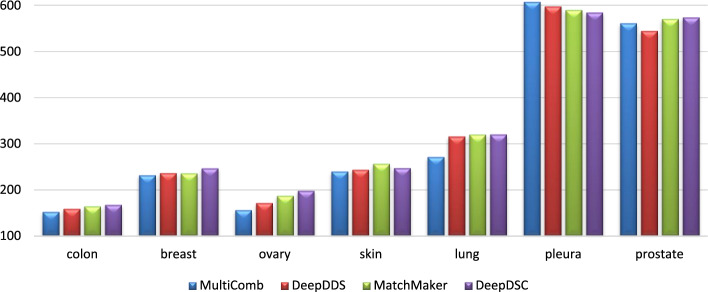
The performance of the MSE for different methods in each 7 tissue types of the cancer cell line

In Fig. 3, the MSE for different models—MultiComb, DeepDDS, MatchMaker, and DeepDSC—is visualized for each unique cell line. It is observed that the MultiComb model has the lowest MSE for most cancer cell lines. In Fig. 4, the MSE for predicting synergy in each cancer tissue across all models did not perform as expected for the ‘pleura’ and ‘prostate’ tissues. This is likely due to the limited data available, as there is only one cancer cell line in the pleura tissue and two in the prostate tissue, which is insufficient for effective learning in those tissues.

**Fig. 5:**
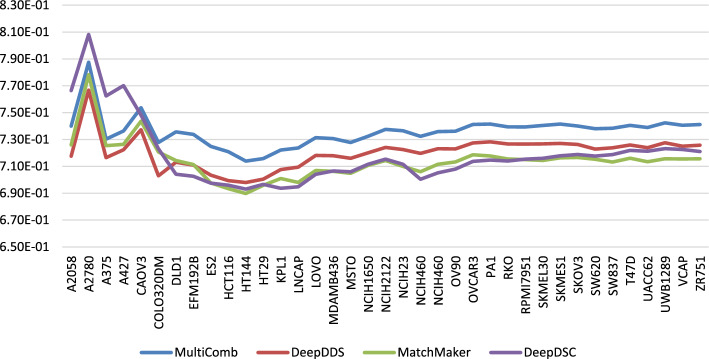
The performance of the ${\text{CC}}_{\text{p}}$ for different methods in each cancer cell line

**Fig. 6:**
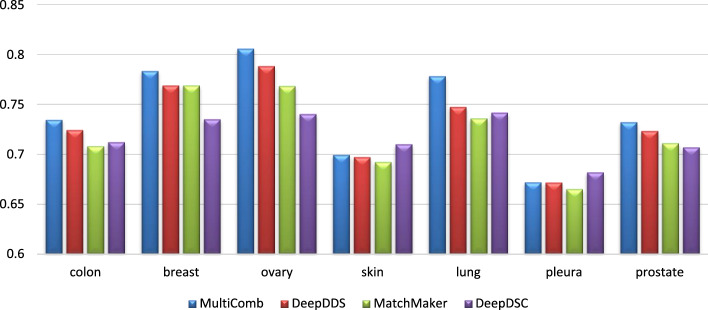
The performance of the ${\text{CC}}_{\text{p}}$ for different methods in each 7 tissue types of the cancer cell line

In Figs. 5 and 6, the ${CC}_{p}$ for all different methods is visualized for each of the 37 cancer cell lines and for each of the 7 cancer tissues, respectively.

Conversely, Table 4 presents the performance MultiComb model in predicting sensitivity scores using the evaluation regression metrics. For the second target score’ sensitivity, MultiComb achieves a low $MSE$ of 15.59 with confidence interval ranges from 13.29 to 17.88. Also, the scores for RMAE and $MAE$ are 3.94 and 2.74, respectively. This indicates that the MultiComb model can predict accurate sensitivity scores.

**Table 4:** The performance of the MultiComb model to predict sensitivity score task

Performance metrics	$MSE$		$RMSE$		$MAE$		${R}^{2}$		${CC}_{p}$		${CC}_{s}$	
	Mean	Std	Mean	Std	Mean	Std	Mean	Std	Mean	Std	Mean	Std
MultiComb	**15.59**	**1.85**	**3.94**	**0.23**	**2.74**	**0.14**	**0.90**	**0.01**	**0.95**	**0.01**	**0.95**	**0.01**

The best-performing results are shown in bold

**Fig. 7:**
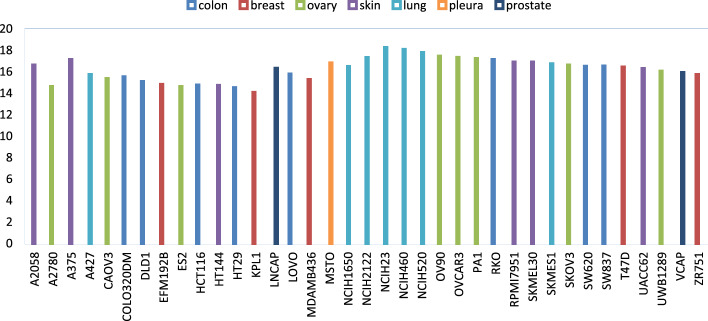
The $\text{MSE}$ of sensitivity prediction score over each cell line

Also, the ${R}^{2}$ score of MultiComb for sensitivity is equal to 0.90 to ensure an acceptable performance model. For the ${CC}_{P}$ metric, MultiComb outperforms 0.95, so the consistency of MultiComb is high. For the last metric, ${CC}_{s}$, the MultiComb achieved a high score of 0.95 in sensitivity to ensure a strong relationship between predicted and actual sensitivity scores. To deeply visualize MultiComb, the sensitivity prediction results are visualized in Fig. 7 according to each cancer cell line. The bar in the figure shows the prediction $MSE$ of each cell whereas the bar color shows the cell line tissue.

**Fig. 8:**
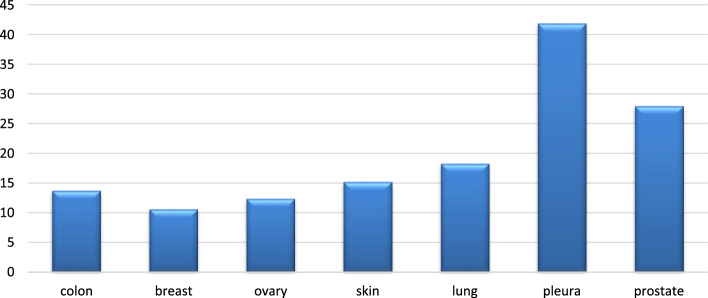
The $\text{MSE}$ of sensitivity score over the 7 tissue types of the cancer cell line

In Fig. 8, the MSE of the MultiComb model’s predicted sensitivity scores for each cancer tissue type is visualized.

**Fig. 9:**
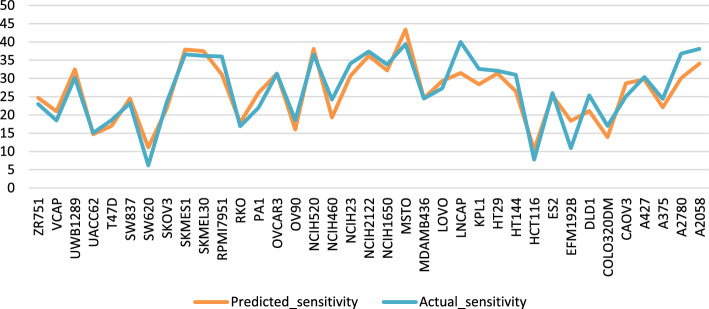
The $\text{MSE}$ of the top predicted and actual sensitivity score for each cancer cell

**Fig. 10:**
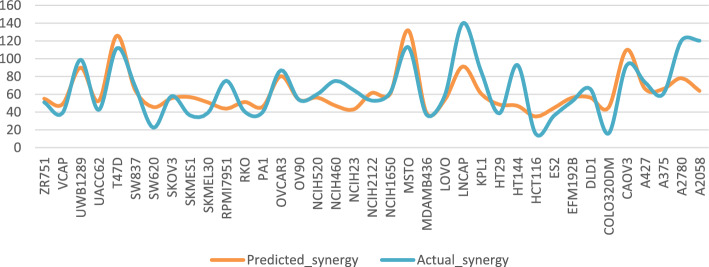
The $\text{MSE}$ of the top predicted and actual synergy score for each cancer cell

The top ten MultiComb predicted sensitivity scores are compared with actual sensitivity scores in Fig. 9. Also, the top predicted synergy scores are compared with actual synergy scores in Fig. 10.

To further analyze the important role that the MTDL plays in drug combinations, the ablation study of four different models is shown in Tables 5 and 6 for sensitivity and synergy scores, respectively.

**Table 5:** The comparison of the MultiComb model’s performance in the ablation study for sensitivity scores

Performance metrics	$MSE$		$RMSE$		$MAE$		${R}^{2}$		${CC}_{p}$		${CC}_{s}$	
	Mean	Std	Mean	Std	Mean	Std	Mean	Std	Mean	Std	Mean	Std
Default Model	17.96	1.53	4.23	0.18	3.00	0.10	0.88	0.01	0.94	0.01	0.94	0.01
With attention only	16.66	1.85	4.08	0.22	2.86	0.13	0.89	0.01	0.94	0.01	0.94	0.01
With task relation only	17.76	1.24	4.21	0.15	2.95	0.14	0.88	0.01	0.94	0.01	0.94	0.01
MultiComb	**15.59**	1.85	**3.94**	0.23	**2.74**	0.14	**0.90**	0.01	**0.95**	0.01	**0.95**	0.01

The best-performing results are shown in bold

**Table 6:** The comparison of the MultiComb model’s performance in the ablation study for synergy scores

Performance metrics	$MSE$		$RMSE$		$MAE$		${R}^{2}$		${CC}_{p}$		${CC}_{s}$	
	Mean	Std	Mean	Std	Mean	Std	Mean	Std	Mean	Std	Mean	Std
Default Model	268.32	43.07	16.33	1.28	10.67	0.58	0.51	0.04	0.71	0.03	0.67	0.02
With attention only	249.41	44.15	15.73	1.37	10.19	0.53	0.54	0.05	0.74	0.03	0.70	0.02
With task relation only	256.69	40.76	15.97	1.25	10.00	0.64	0.53	0.02	0.73	0.01	0.71	0.02
MultiComb	**232.37**	41.24	**15.19**	1.32	**9.59**	0.52	**0.57**	0.04	**0.76**	0.02	**0.73**	0.02

The best-performing results are shown in bold

In the default model, the attention mechanism is replaced with two FC layers to represent the two task features then they are fed to the prediction network. The second model used the attention model only where the outputs are fed to the prediction network directly. Finally, the third model represents the two FC layers as in the default model but before to fed to the prediction network, the cross-stitch is learned to discover the relation between the two tasks.

We find that incorporating the attention mechanism into the MultiComb reduces the $MSE$ for both synergy and sensitivity scores by 7% compared to the default model. Moreover, the ${CC}_{P}$, ${R}^{2}$, and ${CC}_{s}$ for synergy scores improve compared to the default model, with ${R}^{2}$ also improving for sensitivity score. However, ${CC}_{P}$ and ${CC}_{s}$ for sensitivity scores do not show significant improvement, likely due to the smaller range of sensitivity score values compared to the synergy range.

For used relation tasks, only the $MSE$ is minimized from the default model by 4.3% and 1.11% for synergy and sensitivity scores respectively. Also, for synergy score prediction measurement is enhanced but for sensitivity score prediction the improvement fraction is small. Finally, for the MultiComb model when attention and relation tasks mechanisms are combined it states the art over the previous three compared models.

**Fig. 11:**
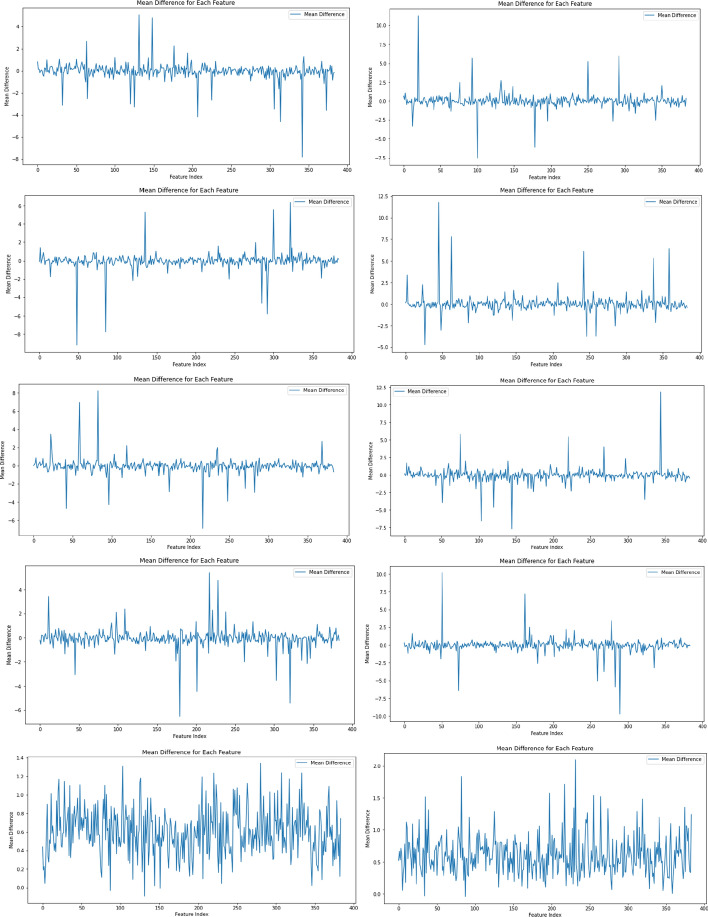
The mean difference between the input features and output features of the attention mechanism is shown. Each row represents a fold in a fivefold cross-validation, with 5 rows in total. The left side shows the mean for the first regression target task (synergy), while the right side shows the mean for the second regression target task (sensitivity)

Furthermore, Fig. 11 illustrates the impact of the attention mechanism on optimizing shared features for each target task across the fivefold cross-validation. This visualization analyzes the differences between the input and output features of the attention mechanism by computing the mean of these differences for each feature across all samples.

The mean difference plot shows the average change in each feature’s value after applying the attention mechanism. Features with higher mean differences indicate significant alterations, potentially highlighting their importance in the learning process. Conversely, features with mean differences near zero suggest minimal changes, implying lower importance.

Table 7 reports the drug combination pairs with the highest predicted synergy scores for each cancer cell line, along with their corresponding predicted sensitivity scores. For example, the pleura cancer cell ‘MSTO’ shows the highest predicted synergy scores with drugs ‘DASATINIB’ and ‘MK-8669’, and also has a high predicted sensitivity score. Conversely, the colon cancer cell line ‘SW620’ has the highest predicted synergy score of 45.6 with drugs ‘VINBLASTINE’ and ‘DASATINIB’ but the predicted sensitivity score is low at 11.1. Therefore, we search for other drug combinations that are more suitable for both sensitivity and synergy scores, such as ‘DASATINIB’ and ‘SUNITINIB’ which have a predicted synergy score of 43.7 and predicted sensitivity score of 35.4.

**Table 7:** The top predicted synergistic drug combinations for each cancer cell line

Issue	Cell_line	Drug1	Drug2	Predicted_Sensitivity	Predicted_Synergy
Breast	ZR751	MK-8669	LAPATINIB	24.7	55.1
Prostate	VCAP	ZOLINZA	LAPATINIB	21	48.6
Ovary	UWB1289	AZD1775	PD325901	32.5	90.1
Skin	UACC62	MK-2206	MK-8669	14.7	52.6
Breast	T47D	METFORMIN	BEZ-235	17	126
Colon	SW837	LAPATINIB	MK-2206	24.5	64.7
Colon	SW620	VINBLASTINE	DASATINIB	11.1	45.6
Ovary	SKOV3	LAPATINIB	MK-8669	21.7	55.6
Lung	SKMES1	PACLITAXEL	ERLOTINIB	37.9	56.9
Skin	SKMEL30	BEZ-235	MK-8776	37.5	50.9
Skin	RPMI7951	MK-8669	BEZ-235	31.1	43.8
Colon	RKO	MK-2206	MK-8669	17.9	51.4
Ovary	PA1	LAPATINIB	BEZ-235	26.1	45.9
Ovary	OVCAR3	LAPATINIB	DASATINIB	31.3	80.5
Ovary	OV90	VINORELBINE	DASATINIB	16	53.8
Lung	NCIH520	DASATINIB	BEZ-235	38.1	56.4
Lung	NCIH460	MK-8669	BEZ-235	19.3	47.2
Lung	NCIH23	MK-8669	BEZ-235	30.7	43
Lung	NCIH2122	DEXAMETHASONE	BEZ-235	36.2	61.6
Lung	NCIH1650	ERLOTINIB	DASATINIB	32.2	60.2
Pleura	MSTO	DASATINIB	MK-8669	43.4	132
Breast	MDAMB436	MK-8669	BEZ-235	24.6	39.1
Colon	LOVO	LAPATINIB	MK-8669	29.3	53.5
Prostate	LNCAP	BEZ-235	MK-8669	31.5	91.4
Breast	KPL1	MK-2206	MK-8669	28.4	60.7
Colon	HT29	DASATINIB	BEZ-235	31.4	48.5
Skin	HT144	MK-8669	BEZ-235	26.5	47
Colon	HCT116	LAPATINIB	BEZ-235	10.5	35
Ovary	ES2	PACLITAXEL	ERLOTINIB	25.3	44.6
Breast	EFM192B	DASATINIB	MK-8669	18.4	56.2
Colon	DLD1	LAPATINIB	DASATINIB	21.1	56.4
Colon	COLO320DM	PACLITAXEL	ERLOTINIB	13.9	45
Ovary	CAOV3	ETOPOSIDE	ERLOTINIB	28.7	110
Lung	A427	DASATINIB	BEZ-235	29.7	66.3
Skin	A375	MK-8669	MK-8776	22.1	65.9
Ovary	A2780	PD325901	DASATINIB	30.1	78.2
Skin	A2058	TEMOZOLOMIDE	MK-8776	34.1	63.8

Furthermore, Table 8 shows the predicted synergy and sensitivity scores of the MultiComb model for samples not included in the O’Neil dataset, obtained from the DrugComb portal [22], and compares these predictions against the actual scores. As observed, the MultiComb model effectively predicts values that are close to the actual scores. While not perfect, the model demonstrates its ability to predict both synergistic and non-synergistic drug combinations accurately.

**Table 8:** Results of the MultiComb model for samples not present in the O’Neil dataset

Cell_line	Drug1	Drug2	Actual sensitivity	Actual synergy	Predicted sensitivity	Predicted synergy
A2058	ETOPOSIDE	GEMCITABINE	38.16	− 6.44	38.89	− 4.46
A375	ETOPOSIDE	GEMCITABINE	27.47	18.88	29.12	− 14.3
RPMI7951	ETOPOSIDE	GEMCITABINE	32.42	− 10.64	37.18	− 5.08
SKMEL30	ETOPOSIDE	GEMCITABINE	9.06	− 0.93	26.32	3.41
UACC62	ETOPOSIDE	GEMCITABINE	21.32	− 17.98	21.20	− 3.38
HCT116	METHOTREXATE	CYCLOPHOSPHAMIDE	43.7	− 25.86	39.52	− 27.35
HT29	METHOTREXATE	CYCLOPHOSPHAMIDE	53.56	− 4.08	23.99	− 4.53
NCIH23	METHOTREXATE	CYCLOPHOSPHAMIDE	10.85	− 33.92	9.02	− 7.81
OVCAR3	METHOTREXATE	CYCLOPHOSPHAMIDE	47.49	− 49.97	18.76	− 29.17
UACC62	METHOTREXATE	CYCLOPHOSPHAMIDE	36.3	− 47.24	22.43	− 2.16
HCT116	PACLITAXEL	CYCLOPHOSPHAMIDE	20.37	− 26.66	20.47	3.6
HT29	PACLITAXEL	CYCLOPHOSPHAMIDE	31.89	− 49.13	28.21	− 3.14
NCIH23	PACLITAXEL	CYCLOPHOSPHAMIDE	16.79	− 31.72	17.55	− 4.32
OVCAR3	PACLITAXEL	CYCLOPHOSPHAMIDE	46.81	− 53.34	22.88	− 10.43
UACC62	PACLITAXEL	CYCLOPHOSPHAMIDE	44.46	− 74.85	23.37	2.17
HCT116	PACLITAXEL	METHOTREXATE	32.59	− 28.2	31.73	− 26.07
HT29	PACLITAXEL	METHOTREXATE	34.43	− 35.35	32.79	− 21.26
NCIH23	PACLITAXEL	METHOTREXATE	22.24	− 14.65	21.41	− 14.81
OVCAR3	PACLITAXEL	METHOTREXATE	42.31	− 8.68	21.47	− 19.01
UACC62	PACLITAXEL	METHOTREXATE	48.35	− 22.13	31.72	− 18.49

Finally, two distinct regression models are trained to predict synergy and sensitivity scores separately. Initially, the models learn drug features from drug molecular graphs and extract cancer cell line features from gene expression data, as discussed in the data representation subsection. These features are then concatenated and fed into the prediction subnetwork, as detailed in the MultiComb model, to predict either the synergy or sensitivity score. As observed from Table 9, the MTDL model outperforms single-task learning. This is because the MTDL model can transfer knowledge and share features between synergy and sensitivity tasks, which does not occur in single-task learning.

**Table 9:** Comparison between results of single task learning and MTDL

Performance metrics	$MSE$		$RMSE$		$MAE$		${R}^{2}$		${CC}_{p}$		${CC}_{s}$	
	Mean	Std	Mean	Std	Mean	Std	Mean	Std	Mean	Std	Mean	Std
Synergy (single _task)	280.15	47.48	16.68	1.39	11.21	0.65	0.49	0.05	0.70	0.04	0.64	0.04
Sensitivity (single _task)	19.63	1.24	4.43	0.14	3.18	0.09	087	0.01	0.93	0.01	0.94	0.01
Synergy (MTDL)	**232.37**	41.24	**15.19**	1.32	**9.59**	0.52	**0.57**	0.04	**0.76**	0.02	**0.73**	0.02
Sensitivity (MTDL)	**15.59**	1.85	**3.94**	0.23	**2.74**	0.14	**0.90**	0.01	**0.95**	0.01	**0.95**	0.01

The best-performing results are shown in bold

Based on the discussion given above, the MultiComb model is effective for predicting target scores for drug combinations as it shows a small prediction error, a robust correlation between actual and predicted values, and good consistency. Additionally, MultiComb predicts scores simultaneously by using the MTDL model.

## Conclusion and future work

This paper has proposed the MultiComb model, an MTDL model designed to predict both the synergy and sensitivity scores of drug combinations simultaneously. MultiComb employs a GCN to learn drug features and fully connected layers to extract cell line features. Initially, the GCN processes the drug data, while the cell line features are derived through fully connected layers. These features are then concatenated and input into an attention mechanism, which generates optimized feature representations for both target tasks. The cross-stitch mechanism is employed to learn the interrelationships between these tasks. Finally, the task-specific features are processed through fully connected layers to output the synergy and sensitivity scores. The performance of MultiComb is evaluated using the O’Neil cancer benchmark dataset, which includes drug combinations and their effects on various cell lines. MultiComb demonstrates high ${R}^{2}$, ${CC}_{P}$, and ${CC}_{s}$ for synergy and sensitivity prediction, and achieves lower $MSE$, $RMSE$, and $MAE$ compared to other deep learning models.

To enhance drug combinations further, additional factors beyond synergy and sensitivity scores should be considered to make informed decisions. For instance, assessing the potential side effects of combining two drugs is crucial. While a combination may exhibit high synergy and sensitivity scores, the presence of adverse effects needs careful evaluation. Moreover, understanding the drug design and interactions between combined drugs can provide insights into effectively regulating the drug combination process. This broader perspective aids in making comprehensive decisions about optimizing drug combinations.

## Data Availability

The datasets analyzed during this current study are obtained from the O’Neil dataset and available at the link [https://github.com/samar-monem/Multicomb/raw/main/data.xlsx] The SMILES of drug data used in this study are extracted from the PubChem website [https://pubchem.ncbi.nlm.nih.gov/] to process the drug features, and the SMILES are transformed into molecular graphs using the freely available chemical informatics package DeepChem [https://deepchem.readthedocs.io/en/latest/api_reference/featurizers.html] Also, the gene expression data of cell lines used in this paper are obtained from [https://depmap.org/portal/] with accession number [PRJNA523380] and file name [CCLE_RNAseq_rsem_genes_tpm_20180929]. The code of the proposed MultiComb model is implemented and available at [https://github.com/samar-monem/Multicomb.git].

## Abbreviations

MTDL: Multi-task deep learning
SMILES: Simplified Molecular-Input Line-Entry
$\text{MSE}$: Mean square error
$\text{MAE}$: Mean absolute error
$\text{RMSE}$: Root means square error
${\text{R}}^{2}$: Coefficient of determination
${\text{CC}}_{\text{P}}$: Pearson correlation coefficient
${\text{CC}}_{\text{s}}$: Spearman correlation coefficient
GCN: Graph convolution network
CCLE: Cancer Cell Line Encyclopedia
FC: Fully connected

## References

[1] Malyutina A, Majumder MM, Wang W, Pessia A, Heckman CA, Tang J. Drug combination sensitivity scoring facilitates the discovery of synergistic and efficacious drug combinations in cancer. PLoS Comput Biol. 2019;15(5):e1006752. 31107860 10.1371/journal.pcbi.1006752 PMC6544320

[2] Torkamannia A, Omidi Y, Ferdousi R. A review of machine learning approaches for drug synergy prediction in cancer. Brief Bioinform. 2022;23(3):bbac075. 35323854 10.1093/bib/bbac075

[3] Lin X, Quan Z, Wang ZJ, Ma T, Zeng X. KGNN: knowledge graph neural network for drug-drug interaction prediction. In: IJCAI International Joint Conference on Artificial Intelligence; 2020.

[4] Chen J, Si YW, Un CW, Siu SWI. Chemical toxicity prediction based on semi-supervised learning and graph convolutional neural network. J Cheminformatics. 2021;13(1):1–16. 10.1186/s13321-021-00570-8 PMC8627024 34838140

[5] Weng Y, Liu X, Li H, Lin C, Liang Y. Drug target interaction prediction via multi-task co-attention. Int J Data Min Bioinform. 2020;24(2):160.

[6] Preuer K, Lewis RPI, Hochreiter S, Bender A, Bulusu KC, Klambauer G. DeepSynergy: predicting anti-cancer drug synergy with deep learning. Bioinformatics. 2018;34(9):1538. 29253077 10.1093/bioinformatics/btx806 PMC5925774

[7] Zhang T, Zhang L, Payne PRO, Li F. Synergistic drug combination prediction by integrating multiomics data in deep learning models. In: Methods in Molecular Biology. Berlin: Springer; 2021. 10.1007/978-1-0716-0849-4_12 32926369

[8] Kuru HI, Tastan O, Cicek E. MatchMaker: a deep learning framework for drug synergy prediction. IEEE/ACM Trans Comput Biol Bioinform. 2021;19:2334. 10.1109/TCBB.2021.3086702 34086576

[9] Preto AJ, Matos-Filipe P, Mourão J, Moreira IS. SynPred: prediction of drug combination effects in cancer using full-agreement synergy metrics and deep learning. 2021; (April). Available from: https://www.preprints.org/manuscript/202104.0395/v1 10.1093/gigascience/giac087 PMC9511701 36155782

[10] Kumar V, Dogra N. A comprehensive review on deep synergistic drug prediction techniques for cancer. Arch Comput Methods Eng. 2021;29:1443.

[11] Liu Q, Xie L. TranSynergy: Mechanism-driven interpretable deep neural network for the synergistic prediction and pathway deconvolution of drug combinations. PLoS Comput Biol. 2021;17(2):e1008653. 33577560 10.1371/journal.pcbi.1008653 PMC7906476

[12] Wang J, Liu X, Shen S, Deng L, Liu H. DeepDDS: Deep graph neural network with attention mechanism to predict synergistic drug combinations. Brief Bioinform. 2022;23(1):bbab390. 34571537 10.1093/bib/bbab390

[13] Ramsundar B, Eastman P, Walters P, Pande V, Leswing K, Wu Z. Deep Learning for the Life Sciences. Massachusetts: O’Reilly Media; 2019.

[14] Kearnes S, McCloskey K, Berndl M, Pande V, Riley P. Molecular graph convolutions: moving beyond fingerprints. J Comput Aided Mol Des. 2016;30(8):595. 27558503 10.1007/s10822-016-9938-8 PMC5028207

[15] Barretina J, Caponigro G, Stransky N, Venkatesan K, Margolin AA, Kim S, et al. The Cancer Cell Line Encyclopedia enables predictive modelling of anticancer drug sensitivity. Nature. 2012;483(7391):603. 22460905 10.1038/nature11003 PMC3320027

[16] Yang W, Soares J, Greninger P, Edelman EJ, Lightfoot H, Forbes S, et al. Genomics of drug sensitivity in cancer (GDSC): a resource for therapeutic biomarker discovery in cancer cells. Nucleic Acids Res. 2013;41(D1):D955. 23180760 10.1093/nar/gks1111 PMC3531057

[17] Veličković P, Casanova A, Liò P, Cucurull G, Romero A, Bengio Y. Graph attention networks. In: 6th International Conference on Learning Representations, ICLR 2018—Conference Track Proceedings; 2018.

[18] Vaswani A, Shazeer N, Parmar N, Uszkoreit J, Jones L, Gomez AN, et al. Attention is all you need. In: Advances in Neural Information Processing Systems; 2017.

[19] Misra I, Shrivastava A, Gupta A, Hebert M. Cross-stitch networks for multi-task learning. In: Proceedings of the IEEE Computer Society Conference on Computer Vision and Pattern Recognition; 2016.

[20] O’Neil J, Benita Y, Feldman I, Chenard M, Roberts B, Liu Y, et al. An unbiased oncology compound screen to identify novel combination strategies. Mol Cancer Ther. 2016;15(6):1155. 26983881 10.1158/1535-7163.MCT-15-0843

[21] Loewe S. The problem of synergism and antagonism of combined drugs. Arzneimittelforschung. 1953;3(6):285. 13081480

[22] Zagidullin B, Aldahdooh J, Zheng S, Wang W, Wang Y, Saad J, et al. DrugComb: an integrative cancer drug combination data portal. Nucleic Acids Res. 2019;47(W1):W43. 31066443 10.1093/nar/gkz337 PMC6602441

